# Air Pollution and Cardiac Arrhythmias: From Epidemiological and Clinical Evidences to Cellular Electrophysiological Mechanisms

**DOI:** 10.3389/fcvm.2021.736151

**Published:** 2021-10-28

**Authors:** Shugang Zhang, Weigang Lu, Zhiqiang Wei, Henggui Zhang

**Affiliations:** ^1^Computational Cardiology Group, College of Computer Science and Technology, Ocean University of China, Qingdao, China; ^2^Biological Physics Group, School of Physics and Astronomy, University of Manchester, Manchester, United Kingdom

**Keywords:** air pollution, arrhythmias, cardiac electrophysiology, epidemiology, review

## Abstract

Cardiovascular disease is the leading cause of death worldwide and kills over 17 million people per year. In the recent decade, growing epidemiological evidence links air pollution and cardiac arrhythmias, suggesting a detrimental influence of air pollution on cardiac electrophysiological functionality. However, the proarrhythmic mechanisms underlying the air pollution-induced cardiac arrhythmias are not fully understood. The purpose of this work is to provide recent advances in air pollution-induced arrhythmias with a comprehensive review of the literature on the common air pollutants and arrhythmias. Six common air pollutants of widespread concern are discussed, namely particulate matter, carbon monoxide, hydrogen sulfide, sulfur dioxide, nitrogen dioxide, and ozone. The epidemiological and clinical reports in recent years are reviewed by pollutant type, and the recently identified mechanisms including both the general pathways and the direct influences of air pollutants on the cellular electrophysiology are summarized. Particularly, this review focuses on the impaired ion channel functionality underlying the air pollution-induced arrhythmias. Alterations of ionic currents directly by the air pollutants, as well as the alterations mediated by intracellular signaling or other more general pathways are reviewed in this work. Finally, areas for future research are suggested to address several remaining scientific questions.

## Introduction

Cardiovascular disease (CVD) is the number one cause of death globally and accounts for more than 17 million deaths annually (1, 2). Air pollution is among the leading risk factors for cardiovascular disease and responsible for ~19% of the total CVD deaths (3, 4). Cardiac arrhythmias, which refer to any types of irregular heartbeats or abnormal heart rates, are a major cause of morbidity and mortality in cardiovascular diseases. Common types of cardiac arrhythmias include sinus node dysfunction, supraventricular tachycardia, atrial fibrillation (AF), conduction disorders, ventricular tachycardia (VT), and ventricular fibrillation (VF). Epidemiological studies have substantiated the association of air pollution with a variety of arrhythmia types (5–7).

Toxicity of pollutants can be significantly modified with their components, which suggest disproportionate contributions of different pollutants (8). Therefore, this review is organized with regard to each pollutant separately. Six common air pollutants, including particulate matter (PM), carbon monoxide (CO), hydrogen sulfide (H_2_S), sulfur dioxide (SO_2_), nitrogen dioxide (NO_2_), and ozone (O_3_). The independent role of each pollutant as well as their potential pathological pathways were explored and emphasized regarding the mechanisms of air pollution-induced arrhythmias.

**Figure 1:**
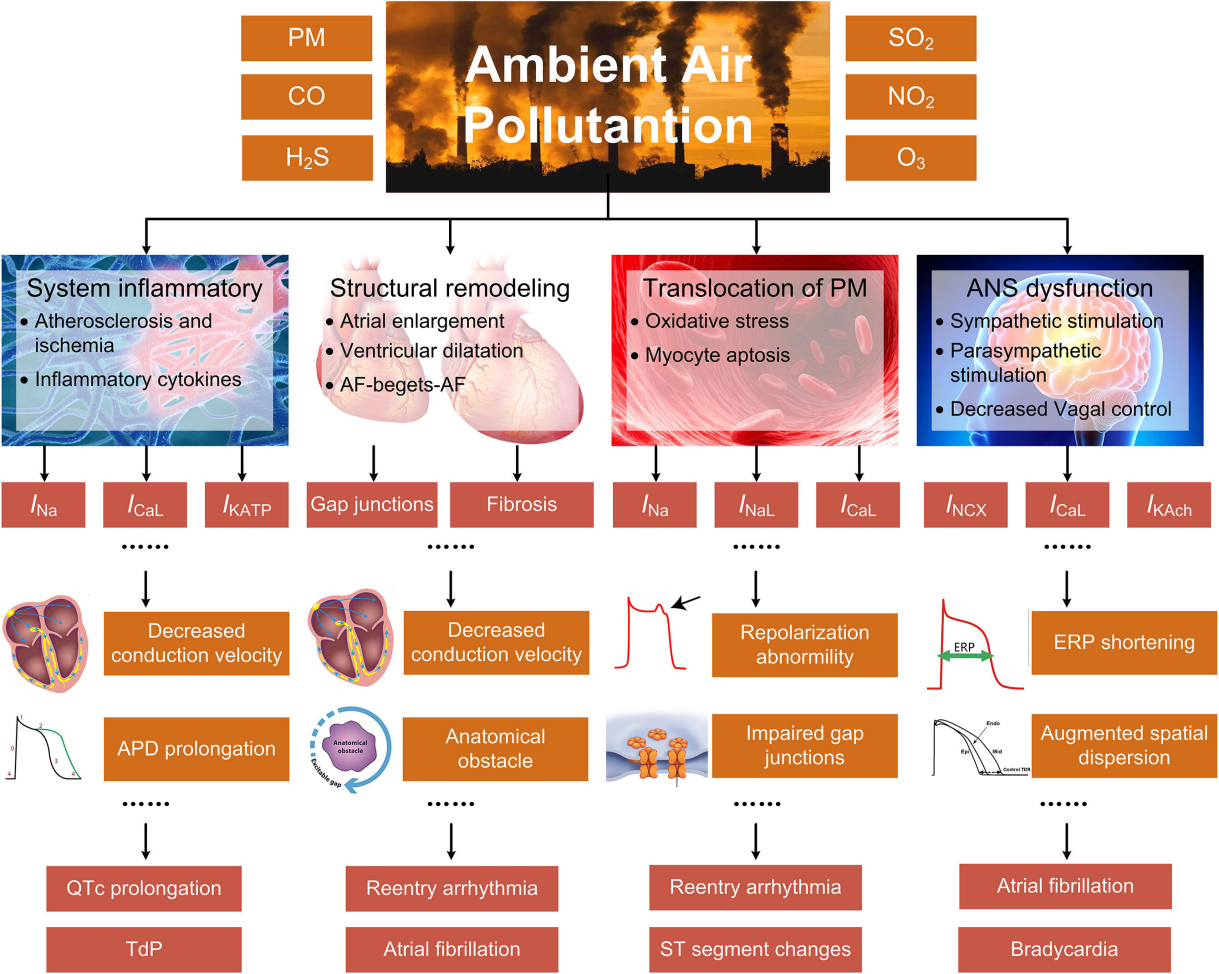
The indirect mechanisms underlying air pollution-induced arrhythmias, including systemic inflammatory response, cardiac structural remodeling, translocation of particles into cardiovascular systems, and ANS dysfunction. The specific pathways are closely linked or overlapped, and only the primary and representative pathways are illustrated for each process.

Systemic inflammatory response, cardiac autonomic dysfunction, cardiac structural remodeling, and translocation of particles into cardiovascular systems are considered the dominant mechanisms underlying air pollution-induced arrhythmias (9). The systemic inflammation and the altered autonomic nervous system (ANS) indirectly cause arrhythmias by a series of actions comprising the progression of atherosclerosis, the increased cytokine levels, and the decreased heart rate variability (HRV), etc. The structural remodeling results in myocardial fibrosis and decreased conduction velocity (CV), and provides substrates for arrhythmias, while the translocated particles can affect ion channel functions through elevating the intracellular levels of reactive oxygen species (ROS). Different from previous surveys that introduced more generalized mechanisms, the present review particularly emphasizes the alterations of cardiac ion channels. This is because most arrhythmia types originate from the dysfunction of ion channels, and minor changes of ionic currents can result in severe cardiac disorder. In the present work, we review both the effects of air pollutants on ion channels that have been proved in experiments and the electrical remodeling that arises as secondary effects of systemic inflammation and other general mechanisms (Figure 1).

## Particulate Matter

Particulate matters (PM) are the materials suspended in Earth's atmosphere in the form of solid particles and liquid drops. There is no strict size boundary within which the particles were considered hazardous. But due to that the larger particles can be filtered by cilia and mucus in the nose and throat, the particles that are <10 μm (PM_10_) in diameter are thought to influence human health as they can be inhaled and get deep into lungs or bloodstream. PM_10_ can be subclassified into coarse PM (PM_2.5−10_), fine PM (PM_2.5_), and ultrafine PM (PM_0.1_) according to the aerodynamic diameter (10). Fine and ultrafine particles derive primarily from the combustion of fossil fuels in industry and traffic (11, 12). Instead, PM_2.5−10_ comes primarily from mechanical processes and is associated with surface or fugitive releases by various human and natural activities (11).

### Epidemiological and Clinical Reports

A growing body of epidemiological evidence demonstrates the associations between PM and cardiovascular diseases (13–15). Among the three size categories of PM, the PM_2.5_ has received the most attention in the past decade. A nationwide study in China suggested a concentration-dependent influence of long-term exposure to PM_2.5_ on cardiovascular health (15). Seniors, rural residents, and non-smokers were shown to be more prone to the adverse effects of PM_2.5_ exposure. It was estimated that each 10 μg/m^3^ decrease in the concentration of PM_2.5_ would avoid over 1.5 million cardiovascular incidents and saved more than 433 thousand lives from cardiovascular diseases annually in mainland China (16). The association between PM_2.5_ and cardiovascular disease is still evident in developed countries where the level of PM_2.5_ is well below the present standard. An investigation conducted in the United States demonstrated that each increase of 10 μg/m^3^ PM_2.5_ was correlated with a 16% increase in mortality from ischemic heart disease, despite the low concentration of PM_2.5_ that below the present 12 μg/m^3^ annual average standard in the United States (17). Though PM_2.5_ is considered to pose the greatest risk to health, there is emerging evidence that coarse particles can exert adverse health influences that are competitive to PM_2.5_ (18). Wang et al. reported that each 10 μg/m^3^ increase of daily PM_10_ was associated with a 1.63% increase in cardiovascular mortality (19). In addition to the size of particles, recent studies confirmed that the toxicity of PM is also related to their constituents, and some components of PM may be more harmful than others. Generally, most studies proved the dominant role of combustion-associated pollutants (20–23). Bell et al. reported that black carbon was one of the most harmful particle types (21). Similarly, a meta-analysis also found a significant association between the black carbon and adverse cardiovascular effects (22). An investigation in Pakistan indicated that exposure to nickel was closely associated with a substantial increase in the risk of cardiovascular diseases (23). As black carbon and nickel were known to be indicators for fossil fuel combustions and industrial emissions, these studies together showed the significant cardiovascular toxicity of combustion-related PM_2.5_. In contrast, constituents related to sea salt showed weak or no association (22, 24).

Cardiac arrhythmia is one of the various cardiovascular events induced by PM. A significant association exists between PM exposure and incidences of various arrhythmia subtypes (25, 26). Atrial fibrillation (AF) is the most common type of arrhythmia induced by PM exposure. A nationwide cohort study concerning the proarrhythmic effects of long-term exposure to PM reported that 10 μg/m^3^ increments of PM_2.5_ were associated with a 17.9% increase of AF, and 10 μg/m^3^ increments of PM_10_ was associated with a 3.4% increase (27). A recent epidemiological study conducted in Canada demonstrated that long-term exposure to PM_2.5_ was in association with the occurrence of AF even at a very low concentration level (28). A correlation was also found between short-term PM exposure and increased risks of AF. An investigation based on patients with dual-chamber implantable cardioverter-defibrillators (ICDs) suggested significant effects of PM_2.5_ on triggering AF. The odds of AF increased by 26% for each 6 μg/m^3^ increase in PM_2.5_ in the 2 h prior to the onset of AF (29). For healthy individuals, Lee et al. demonstrated that short-term exposure to PM_2.5_ was associated with AF in the general population with no AF history (30). Similar findings were also reported in other investigations (5, 31). Besides atrial arrhythmias, PM was also associated with ventricular arrhythmias. An investigation based on high-risk populations with implantable cardioverter-defibrillators (ICDs) or cardiac resynchronization therapy defibrillators (ICD-CRT) showed a positive and significant correlation between short-term exposure to PM and ventricular arrhythmias including VT and VF (32). The study also showed that patients who had previous myocardial infarction were more prone to PM-induced ventricular arrhythmias (32). Tsai et al. reported that PM_2.5_ exposure was associated with high ventricular premature complex (VPC) burden in patients without structural heart disease (33). The VPC burden is one of the clinical indicators of decreased cardiac function in continuous ECG monitoring (34, 35). It measures the percentage of ectopic beats in all heartbeats, and a higher VPC burden is associated with increased risks of cardiovascular mortality (36).

### Potential Proarrhythmic Mechanisms

**Figure 2:**
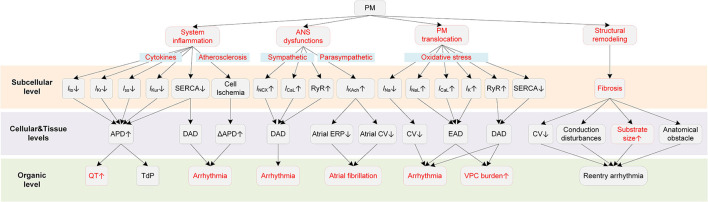
Potential mechanisms underlying PM-induced arrhythmias. Red boxes indicate effects of PM that have been explicitly demonstrated.

It is generally considered that PM-induced cardiac arrhythmias are triggered by several major mechanisms, including inflammatory responses, ANS dysfunctions, direct effects of translocated PM into circulation, and cardiac structural remodeling (9, 37) (Figure 2).

PM can exert proarrhythmic effects indirectly that are mediated through the systemic inflammatory responses (38–40). Systemic inflammation is known to contribute to the progression of atherosclerosis (41), resulting in chronic and acute ischemia. Ischemic heart disease leads to ion channel remodeling, augments spatial dispersion of depolarization, thereby providing substrates for cardiac arrhythmias (42, 43). Second, in some severe inflammatory states such as sepsis and septic shock, the increased cytokine levels cause myocardial dysfunction that might contribute to the occurrence of various types of arrhythmias. Besides, the inflammation-related coagulation response might also act as an indirect factor for arrhythmogenesis (38). Inflammatory cytokines can also directly modulate the function of ion channels and calcium homeostasis (44). For example, tumor necrosis factor (TNF), as one of the inflammatory cytokines, could reduce the sarcoplasmic/endoplasmic reticulum Ca^2+^ ATPase (SERCA), leading to increases in intracellular Ca^2+^ levels and risks of arrhythmogenesis (45). Recent evidence from clinical studies proved the direct influences of inflammatory cytokines on potassium currents, suggesting inflammation as a novel risk factor for QT-syndrome and Torsade de Pointes (TdP) (46, 47). Lazzerini et al. showed that systemic inflammation could directly prolong the corrected QT (QTc) interval via the cytokine-mediated ventricular electrical remodeling effects (48). Overall, these findings together reveal the role of inflammation in arrhythmia pathogenesis and point out one of the potential explanations for the PM_2.5_-induced cardiac arrhythmias.

Another indirect proarrhythmic pathway is the ANS dysfunction (49). PM is documented to be in relation with a decreased HRV (50, 51), which is an indicator of cardiac autonomic dysfunction and is a risk factor for fatal ventricular arrhythmias (52). Liao et al. observed that the magnitude of association between PM_2.5_ and AF was greatly attenuated after adjusting for cardiac autonomic modulation among healthy people, suggesting that ANS was partly responsible for the PM-induced cardiac electrophysiological changes (53). ANS can be subdivided into two antagonistic sets of nerves, i.e., the sympathetic nervous system, and the parasympathetic nervous system. The sympathetic stimulation in normal hearts increases the heart rate by enhancing the funny current (*I*_f_), shortens APD, and reduces transmural dispersion of repolarization (54, 55). In contrast, in pathological states induced by PM, the dysregulated sympathetic stimulation might enhance the dispersion of repolarization (56, 57) and promotes afterdepolarization (58, 59). Specifically, the β-adrenergic receptor stimulation activates the stimulatory Gs proteins, which stimulates the adenylyl cyclase that catalyzes the conversion of adenosine triphosphate (ATP) to cyclic adenosine monophosphate (cAMP). The cAMP then activates the protein kinase A (PKA) and the latter causes the phosphorylation of the L-type calcium current (*I*_CaL_) and ryanodine receptors, leading to excessive calcium influx and sarcoplasmic reticulum (SR) Ca^2+^ release. Elevated intracellular Ca^2+^ activates the Na^+^/Ca^2+^ exchanger current (*I*_NCX_) and finally induces delayed afterdepolarization (DAD) in myocytes (60, 61). Computer simulations further revealed that the SR Ca^2+^ overload and release can be produced synchronously and finally lead to ectopic beats (62). While it seems apparent that the vagal stimulation, as opposed to sympathetic stimulation, is antiarrhythmic by mitigating the influences of its counterpart, it can be proarrhythmic in the atria due to its different effects in atria and ventricles (54). In detail, the vagal activation prolongs APD and ERP in the ventricle (63, 64), whereas it reduces the atrial ERP, augments the spatial electrophysiological heterogeneity, and promotes EAD (54). Such difference is partly attributable to an inwardly rectifying potassium current abundant in the atria, i.e., *I*_KAch_. *I*_KAch_ is activated by the parasympathetic neurotransmitter acetylcholine (Ach), and the activation of *I*_KAch_ under the condition of vagal stimulation substantially shortens APD. Besides, simulations show that the *I*_KAch_ can result in the hyperpolarization of the resting membrane potential, which in turn slows the conduction velocity in the atria (65). The effect of conduction slowing can be further enhanced by fibrosis—a condition that widely occurred in AF patients. The shortened APD and decreased CV induced by *I*_KAch_ together contribute to a reduced wavelength for reentry and therefore increase the susceptibility to atrial arrhythmias (65). Above all, either sympathetic or parasympathetic dysfunctions can be proarrhythmic, with afterdepolarization acting as triggers while shortened refractory period and augmented spatial dispersion of repolarization acting as substrates for arrhythmogenesis.

Though the proarrhythmic effect of PM on cardiac electrical activity can be secondary to the systemic inflammatory process, in some instances, some small PM such as ultrafine particles can cross the pulmonary epithelium and penetrate into the circulation, leading to direct influences on the cardiovascular system (66, 67). Miller et al. observed that nanoparticles could translocate from the lung to the circulation, and these nanoparticles were found accumulated at sites of vascular inflammation in both mice and humans (68). These translocated particles can directly alter the membrane ion channels and the cellular electrophysiology of myocytes. Savi et al. reported that the nanoparticles directly entered ventricular cardiomyocytes, resulting in shortened action potential duration (APD) and effective refractory period (ERP), and also an increased membrane excitability (69). These effects together provided substrates for electrical alternans and increased the propensity to arrhythmias. It was also reported that the intracellular ROS formation was increased by 16% after only 1-h exposure, which partially accounted for the impaired channel functions (69). Indeed, oxidative stress was proved to be associated with arrhythmias via various actions (70, 71). In terms of ionic effects, oxidative stress was recorded to affect all major ionic currents (71). For sodium channels, the elevated ROS enhanced the late sodium current (*I*_NaL_) (72) and decreased the sodium current (*I*_Na_) (73). Such effects resulted in the occurrence of early afterdepolarization (EAD) and the decreased CV, which facilitated the formation of reentry arrhythmia by providing both the trigger and the substrate. Besides, ROS can also stimulate *I*_CaL_ (74) and modulate intracellular Ca^2+^ handling through influencing ryanodine receptors and SERCA (75–78). These effects can lead to abnormal depolarization and induce triggered activity. Specifically, ROS decreases the repolarization reserve by stimulating *I*_CaL_, which facilitates the formation of EAD (74). Besides, the elevated ROS oxidases and enhances the Ca^2+^/calmodulin-dependent kinase II (CaMKII), and the latter then phosphorylates and activates the ryanodine receptor (79, 80). The ryanodine receptor is known to act a physiological role in a biological process named “calcium-induced calcium release (CICR)”, whereas the oxidative stress-induced activation of the ryanodine receptor may lead to Ca^2+^ leak (79). ROS can also inhibit SERCA. SERCA is responsible for the calcium transportation from the cytosol back into the SR during diastole, and the inhibition of SERCA hinders the SR Ca^2+^ reuptake (81). Above two effects of ROS together contribute to overloaded intracellular Ca^2+^. The overloaded Ca^2+^ is expelled in exchange for Na^+^ via Na^+^/Ca^2+^ exchanger, thereby forming *I*_NCX_ and may triggering delayed afterdepolarizations (DADs). For potassium channels, ROS was reported to suppress the ATP-sensitive potassium current (*I*_KATP_) (82), the transient outward potassium current (*I*_to_) (83), the rapid delayed rectifier potassium current (*I*_Kr_) (84), and the slow delayed rectifier potassium current (*I*_Ks_) (84), which led to a prolonged APD and the occurrence of EAD. Besides the ionic currents, ROS can also change the cell coupling by promoting myocardial fibrosis (85) or impairing gap junctions (86). Taken together, translocated particles can directly influence cardiac electrophysiological activities by affecting various ion channels, and ROS might be an important mediator in these actions.

Finally, PM_2.5_ can also exert proarrhythmic effects by promoting cardiac structural remodeling. An epidemiological study reported that exposure to PM_2.5_ was significantly associated with cardiac ventricular dilatation including larger left ventricular end-diastolic and end-systolic volumes, and right ventricular end-diastolic volume (87). The remodeling effect of PM_2.5_ was also confirmed in experimental studies (88–90). The structural remodeling induced by air pollutants can promote the development of the AF and the lethal VF. In detail, tissue fibrosis slows the conduction velocity while increases the heart dimension (91), resulting in an increased substrate size for reentry arrhythmias (92). Fibrosis also promotes arrhythmias by interrupting the fiber bundle continuity, leading to local conduction disturbances or anatomical reentry (93). The induced arrhythmias in turn cause more serious cardiac remodeling and abnormalities, forming an auto-reinforcing process such as ‘AF-begets-AF' (91, 94, 95). Computer simulations substantiate the association between structural remodeling caused by PM exposure and cardiac arrhythmias and provide insight into the mechanisms. Most recently, Palacio et al. modeled the diffuse myocardial fibrosis, a type of structural remodeling caused by exposure to ambient PM, in a 3D model of human atria (96). The simulation results suggested that the PM-induced diffuse fibrosis reduced the CV and increased the reentrant fibrillary dynamics with the increase of the fibrosis density.

## Carbon Monoxide

Carbon monoxide (CO) is a hazardous air pollutant presented particularly in heavily polluted urban areas. It is colorless and odorless, and primarily arises from incomplete combustion of hydrocarbon sources. Exposure to environmental CO such as road traffic has shown to be associated with poor cardiovascular outcomes (97).

### Epidemiological and Clinical Reports

CO is among the most frequently studied gaseous pollutants (98). A meta-analysis based on 9 million people in the United States established the correlation between the ambient CO exposure and heart failure that 1 ppm increment of CO was associated with a 3.52% increase in heart failure hospitalizations or mortality (98). A recent study reported that ischemic heart disease deaths increased 21.1% for every 10 μg/m^3^ increase in CO (99). Accidental exposure to higher levels of CO is not rare in industrial settings or common road traffic environments, which could lead to ECG changes and arrhythmias. A case-control study by Hanci et al. reported that 5 of 30 acute CO poisoning patients had arrhythmias, with one of them had ventricular arrhythmias and the other four had atrial arrhythmias (100). Among those patients of atrial arrhythmias, three of them were supraventricular tachycardia and the other one had atrial extrasystoles. Akdemir et al. reported that a patient with no history of heart diseases and arrhythmias developed AF after CO exposure (101). Gedela et al. reported a similar case for CO-induced AF (102). Effects of CO on cardiac electrophysiology can be manifested on the ECG as ST-T changes, prolonged QT intervals, increased QT dispersion durations, and increased P-wave dispersion durations. ST-segment changes are among the most frequently observed ECG manifestations of CO poisoning, and various abnormal ST-T morphologies including ST-segment elevation, ST depression, and T-wave inversion have been reported in CO poisoning patients (103–105). Frequently observed ST-segment changes indicate that the myocardium is prone to be injured in CO poisoning, and a previous clinical investigation showed that up to 37% of the enrolled patients developed myocardial injury after CO exposure (103). As for the QT changes, Hanci et al. reported an average QTc interval of 429.6 ms in the CO poisoning group among which 30% had QTc intervals above 440 ms. The average QTc interval in the control group, in contrast, was only 385.6 ms and none of them had extended QTc (100). In a recent clinical case, a QTc interval of up to 622 ms was observed (106). In addition to the QT prolongation effect, CO was also reported to significantly increase the dispersions of both QT intervals (QTd) and P-waves (Pwd), and the Pwd was almost doubled in the CO group (106).

### Potential Proarrhythmic Mechanisms

**Figure 3:**
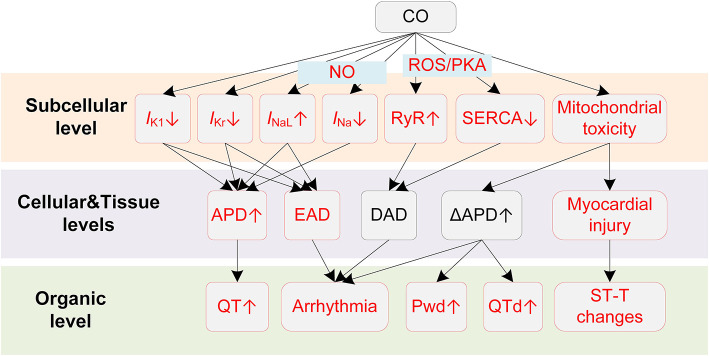
Potential mechanisms underlying CO-induced arrhythmias. Red boxes indicate effects of CO that have been explicitly demonstrated.

Potential mechanisms of CO-induced arrhythmias are summarized in Figure 3. CO has been proven to have mitochondrial toxicity. The high binding affinity of CO to intracellular myoglobin in the myocardium would impair the oxygen delivery to the mitochondria, causing subsequent energy crisis and cardiac contractile decrease (107). CO could also impair the mitochondrial respiratory chain via inhibiting cytochrome C oxidase, causing decreased glutathione concentrations and declined ATP generation (107). In addition to the intracellular influences, CO also affects multiple membrane channels. For the calcium channel, Scragg et al. found that CO suppressed the *I*_CaL_ (108). The inhibition effects occurred via an increase in ROS production specifically from mitochondria. In settings of chronic exposure to environmentally relevant CO levels, the alterations of *I*_CaL_ were not consistent with above findings. Specifically, CO was observed to increase *I*_CaL_, and such influences were only observed in epicardial cells (109). Though the uneven changes of the *I*_CaL_ might alter the transmural repolarization dispersion and potentially provide a substrate for ventricular arrhythmias, this study considered it to have limited impact (109). Instead, the study attributed the arrhythmogenic effect of chronic CO exposure to Ca^2+^ overload. Particularly, the intracellular Ca^2+^ overload results from the reduced SERCA expression and the PKA-phosphorylation of ryanodine receptors. The impairment in the Ca^2+^ reuptake by SR, and the Ca^2+^ leak in SR, together contribute to the Ca^2+^ overload. The overloaded Ca^2+^ in turn activates *I*_NCX_ and leads to DAD (109). In addition to calcium currents, sodium currents including *I*_Na_ and *I*_NaL_ could also be affected by CO. Dallas et al. showed that CO augmented *I*_NaL_ through the NO-mediated pathway (110). CO was able to activate the synthesis of NO, and the elevated NO level consequently led to nitrosylation of the Na_v_1.5 channel protein, causing the augmentation of *I*_NaL_. The above effect of CO led to EAD-like Ca^2+^ transients. In the circumstances of additional stress (mimicked by injecting β-adrenergic agonist isoprenaline), CO-induced EADs could easily lead to VF and sudden death (110). In contrast to the enhanced *I*_NaL_, CO was reported to inhibit the *I*_Na_, and the inhibition was also NO-dependent (111). The inhibition effect was associated with a hyperpolarization shift of steady-state inactivation properties of channels. Given the fact that the inhibition of *I*_Na_ can lead to Brugada syndrome-like arrhythmias (112), the study suggested a new potential proarrhythmic mechanism of CO (111). Finally, for potassium channels, CO decreases the rapidly activating delayed rectifier potassium current (*I*_Kr_) (113) and the inwardly rectifying potassium channel current (*I*_K1_) (114). The former was observed in guinea pigs and HEK293 cells and was mediated by the formation of NO and peroxynitrite (113). Liang et al. suggested that the prolonged APD of rat myocytes arouse from the combined effects of CO on *I*_NaL_ and *I*_K1_, and the ratio of APD prolonging by *I*_NaL_ and *I*_K1_ was about 4:3. In addition to above experimental observations about the alteration in ionic currents upon exposure to CO, it should be noted that CO can also indirectly contribute to the cellular electrophysiological remodeling via myocardial ischemia. Under the ischemic condition, intracellular acidosis activates the Na^+^/H^+^ exchanger to exclude hydrogen ions in exchange for sodium ions, and the elevated sodium is then exchanged for calcium through the Na^+^/Ca^2+^ exchanger, which finally leads to intracellular Ca^2+^ overload (115, 116). Acidosis also inhibits *I*_CaL_, and this effect results in APD shortening together with the reduced *I*_KATP_ by hypoxia (115). Besides, the ischemia is also accompanied by hyperkalemia. The elevated extracellular potassium results in depolarization of the resting potential, which then partially or completely inactivates sodium channels but activates the late sodium channel (115, 117).

Above experiments regarding the alterations of ion channels by CO provided the cellular basis for the clinically observed ECG changes and CO-induced arrhythmias. The myocardial injury that comprises myocardial ischemia and infarction is the direct cause of the ST-T change, and it is also the primary factor responsible for the CO-induced arrhythmias (97). Depending on the injured location and the time of injury, the MI-induced ST-T changes can be very different. In the case of subendocardial infarction, as a common clinical type, the phase-2 of the endocardial action potential is lower than the healthy epicardial myocytes, which is manifested as the depressed ST-segment on the ECG. The involvement of *I*_KATP_ and the decreased *I*_CaL_ by acidosis under ischemia condition shortens the endocardial APD, and therefore changes the transmural repolarization sequence, causing an inverted T-wave. In contrast, the transmural or subepicardial infarction types are manifested as enhanced ST-segment. Arrhythmogenesis substrates can be formed in either case, as the spatial dispersion of repolarization increases the risk of developing unidirectional block when the premature stimulus (i.e., EAD, DAD) occurs. Furthermore, the ischemia-induced cellular electrical remodeling also contributes to repolarization heterogeneities, especially near the ‘border zone' of infarction (118).

In addition to myocardial injury-related ST-T changes, CO also results in QT changes that might be achieved through distinct pathways other than ischemia-induced ion channel remodeling. The QT interval on the ECG corresponds to the time it takes for heart ventricles to depolarize and repolarize, and as the depolarization is quite fast, the repolarization phase accounts for most of the QT duration. A prolonged QT interval on the ECG indicates that the ventricle repolarization is delayed, or from a cellular perspective, the duration of AP repolarization is prolonged. In this regard, the observed QT prolongation in patients with CO poisoning can be attributed to the impaired repolarization reserve resulted from several altered ion channels, i.e., increased *I*_NaL_ (110), decreased *I*_Kr_ (113), and decreased *I*_K1_ (114). Prolonged QT or QTc durations increase the risk for polymorphic VT, VF, TdP, and even sudden death (119, 120). In this regard, Hanci et al. found a positive correlation between COHb level and QTc duration, providing a rationale between acute CO poisoning and the development of ventricular arrhythmia (100). Noted that the prolonged QTc was also observed when people were exposed to traffic-related air pollutants (121). Therefore, CO might be a major role in traffic-related pollution-induced heart diseases, exerting independent detrimental influences on cardiovascular systems. Another observed manifestation of QT changes by CO is the increased QTd. The QTd reflects the transmural heterogeneities of repolarization time within ventricles, and the heterogeneities are secondary to regional differences in APD and activation time (122). Recent simulation studies suggested that the increased transmural dispersion of repolarization (TDR) by CO was attributed to the more prolonged APD in midmyocardial cells comparing to epicardial or endocardial cells (123, 124), and the different APD changes may arise from the heterogeneous current densities in different cell types (125). The increased TDR then contributes to higher risks of developing unidirectional conduction block (measured as the vulnerable window), and finally leads to TdP, reentry arrhythmias, and VF (126). Extensive investigations have reported that QTd was significantly increased in CO intoxicated patients (100, 127). Therefore, the enhanced repolarization dispersion is another potential proarrhythmic factor of CO.

The CO-induced ECG manifestations and arrhythmias related to the atria also have their cellular basis. CO was observed to increase the P-wave dispersion (Pwd), which is a non-invasive indicator of AF. The Pwd is measured as the difference between the maximum and minimum P-wave durations on the ECG, and it reflects the inhomogeneous and discontinuous propagation of sinus impulses through the atria. Similar to QTd, the increased Pwd is secondary to the increased heterogeneity of electrophysiological properties within the atrial myocardium (128). In the case of CO, the clinically observed Pwd may originate from several ionic remodeling effects. First, CO could decrease the conduction velocity of excitation wave (123, 124); therefore the intra- and interatrial conduction times of sinus node impulses can be prolonged. Such assumption is based on the fact that CO could inhibit *I*_Na_ (111). As *I*_Na_ is a prominent factor in determining the conduction velocity in cardiac tissues, its inhibition will delay the propagation of excitation in the atria. In addition to the conduction properties, CO may also contribute to the regional heterogeneity by differently prolonging the APD in a dose-dependent way (124). The increased heterogeneity provides substrates for atrial arrhythmias by increasing the likelihood of unidirectional conduction block, while the CO-induced EAD (via increased *I*_NaL_) (110) and DAD (due to the Ca^2+^ overload) (109) act as triggers in initiating atrial arrhythmias, providing that the afterdepolarization activities were able to overcome the source-sink effect and form an ectopic beat (129, 130). These cellular mechanisms provide insights of the clinical observed Pwd changes and atrial arrhythmias.

## Hydrogen Sulfide

Hydrogen sulfide (H_2_S) is a colorless air pollutant with a strong odor of rotten eggs. It can be largely produced in industrial activities, such as food processing, coke ovens, and petroleum refineries.

### Epidemiological and Clinical Reports

Acute H_2_S poisoning often occurs in occupational exposures. H_2_S is immediately fatal when it reaches concentrations of over 500–1,000 ppm (131). For environmentally relevant H_2_S concentration, an investigation based on the naturally H_2_S-exposed population showed that cardiovascular was one of the adverse health effects of chronic H_2_S exposure (132). Another study that focused on the short-term effects of low-level H_2_S exposure showed that H_2_S exceeding 7 μg/m^3^ was associated with admission and emergency department visits with heart disease (133). The study also concluded that males and old people were more susceptible to H_2_S. Available clinical reports of H_2_S poisoning demonstrated consistent cardiac changes, i.e., ST-segment elevation and malignant arrhythmias. Specifically, in an acute H_2_S exposure case, the patient was reported to have episodes of VT two days after exposure, and the course was complicated by other observations including ST-segment elevation, hyperkalemia, acidosis, etc. The patient died on the third day in the hospital (134). The ST-segment elevation was also reported in another clinical study (135). The patient was exposed to 20.3~25.6 mg/m^3^ (20.3~25.6 ppb) H_2_S but showed no obvious ECG changes on the first day of admission; however, subsequent significant ST-segment elevation along with changes in markers of myocardial injury was observed on the fourth day of hospitalization (135). Another recent case presented identical cardiac changes and clinical course, where the patient was free of ECG abnormalities on the first day of exposure, but showed extensive elevation of ST-segment and dramatic increase of myocardial enzyme index two days after exposure. The patient then developed VF on the next day (136). It can be easily observed from above cases that patients with H_2_S poisoning usually presented no clinical symptoms or ECG changes immediately after exposure, but could experience ST-segment elevation and develop lethal arrhythmias after 2~4 days. The delayed and abrupt onset of cardiac changes are rather dangerous and suggest the insidious effects of H_2_S on the myocardium.

### Potential Proarrhythmic Mechanisms

**Figure 4:**
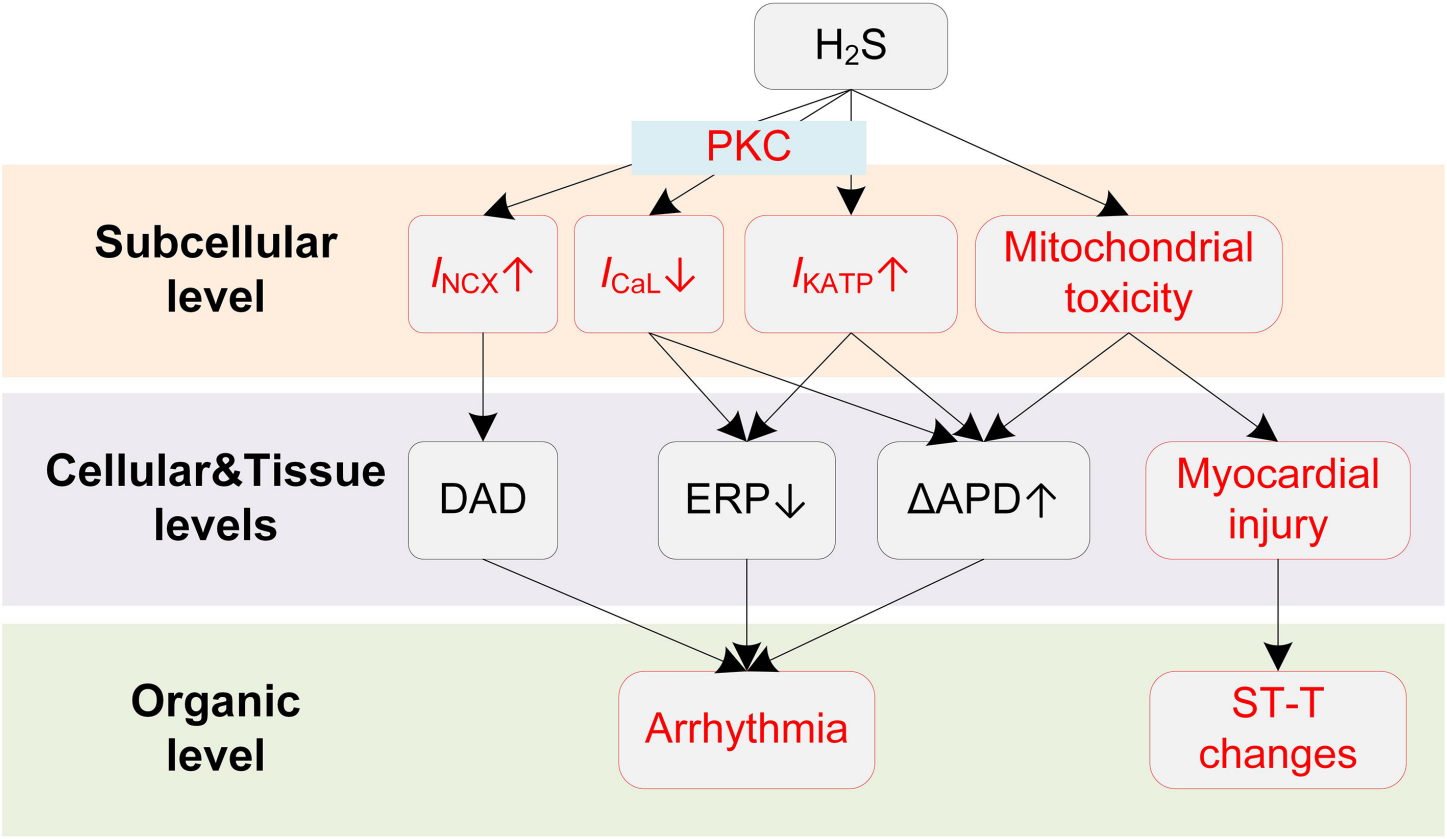
Potential mechanisms underlying H_2_S-induced arrhythmias. Red boxes indicate effects of H_2_S that have been explicitly demonstrated.

Potential mechanisms of H_2_S-induced arrhythmias are illustrated in Figure 4. The clinically observed ST-segment changes on the ECG suggest the occurrence of myocardial injury when exposed to H_2_S. Similar to CO, H_2_S causes myocardial damages due to its mitochondria toxicity. Specifically, H_2_S was reported to inhibit cytochrome C oxidase (137), leading to the blockade of the electron transport in the respiratory chain and the utilization of molecular oxygen (135, 138). The damaged respiratory chain could subsequently result in disrupted aerobic metabolism of cells and impaired ATP production, and ultimately leads to reduced oxygen use of cardiomyocytes and myocardial damage (136). Large dispersion in repolarization can be formed under myocardial injury conditions, e.g., in the border zone of the regionally ischemic heart or near the infarcted tissue area (139), and thereby increases the likelihood of induction of unidirectional block and arrhythmias.

While the repolarization dispersion provides a plausible explanation for the frequently observed malignant arrhythmias, the proarrhythmic effects of H_2_S can be further exaggerated by its actions on cardiac cellular electrophysiology. Specifically, hypoxia is known to activate the *I*_KATP_ due to a dropped ratio of adenosine triphosphate (ATP) and adenosine diphosphate (ADP), and H_2_S is also proved to be a KATP channel opener (140). The activation of *I*_KATP_ can be proarrhythmic as it accelerates the phase 3 repolarization and reduces APD and ERP, leading to shortened critical length for initiating reentry arrhythmias (141). H_2_S was also reported to inhibit the *I*_CaL_ in a concentration-dependent manner (142, 143), and the involvement of *I*_CaL_ was further evidenced by the observation that blockage of *I*_KATP_ did not completely eliminate the effect of H_2_S (144). In our previous simulation study, we investigated the proarrhythmic effects of H_2_S using a multi-scale virtual heart (43). Briefly, the effects of H_2_S on ionic currents were incorporated into a mathematical myocyte model, and were further expanded to tissue levels. The simulation results suggested that H_2_S could decrease the spatial critical length of initiating arrhythmias, augment transmural repolarization dispersion, and depress cell excitability. These actions together lead to increased tissue susceptibility for initiation and maintenance of reentry arrhythmia. In addition, the vulnerability to reentry arrhythmia could be further exacerbated in mild ischemia due to the involvement of *I*_KATP_ (43).

The mechanism of proarrhythmic effects of H_2_S can be more complicated in the atria. Recently, Chan et al. performed a comprehensive investigation on the influences of H_2_S on the atria and provided novel insights into the proarrhythmic effects of H_2_S (145). The study reported that NaHS (H_2_S donor) significantly reduced sinoatrial node (SAN) beating rates, causing SAN dysfunction and potentially the AF. In pulmonary vein myocytes, NaHS increased *I*_KATP_ and *I*_NCX_. The activation of *I*_NCX_ could potentially lead to DADs, acting as triggers for arrhythmogenesis. These effects were mediated by the protein kinase C (PKC) as they were attenuated by PKC inhibitors. The PKC-mediated increases in *I*_KATP_ and *I*_NCX_ were also observed in atrial myocytes; however, such effects were only presented in left atrial myocytes but not right atrial myocytes. This interesting observation demonstrates that H_2_S can lead to increased interatrial dispersion, and therefore increases the risk of supraventricular arrhythmias (145). Noted that PKC may also regulate other channels in addition to *I*_KATP_ and *I*_NCX_, such as *I*_to_ (146), *I*_NaL_ (147), *I*_Ks_ (148), and their potential roles in H_2_S-induced arrhythmias warrants further investigation.

## Sulfur Dioxide

Sulfur dioxide (SO_2_) is a colorless gaseous pollutant with a pungent odor. SO_2_ in the air comes mainly from the burning of fossil fuels contaminated with sulfur compounds or from copper smelting. It can also be released naturally from volcanic eruptions.

### Epidemiological and Clinical Reports

Epidemiological studies have demonstrated the correlation between SO_2_ and cardiovascular events. A nationwide study in China demonstrated that SO_2_ was associated with increased total and cardiorespiratory mortality (149). Amsalu et al. reported that the increased SO_2_ concentration was associated with multiple cardiovascular diseases, including coronary heart disease, AF, and heart failure (150). Specifically for arrhythmias, Zhao et al. reported that a 10 μg/m^3^ increase of daily SO_2_ led to a 2.07% increase of outpatient arrhythmia visits, and the influences were stronger in older people and in females (151). A recent epidemiological study showed that SO_2_ was significantly associated with arrhythmias (152). The proarrhythmic effect of SO_2_ was significant in the middle-aged population and was primarily observed in the cold season (152). The cardiovascular influences were also observed after long-term SO_2_ exposure. An investigation conducted in South Korea suggested that long-term exposure to a high level of SO_2_ was consistently and significantly associated with an increased ischemic heart disease mortality (153).

### Potential Proarrhythmic Mechanisms

**Figure 5:**
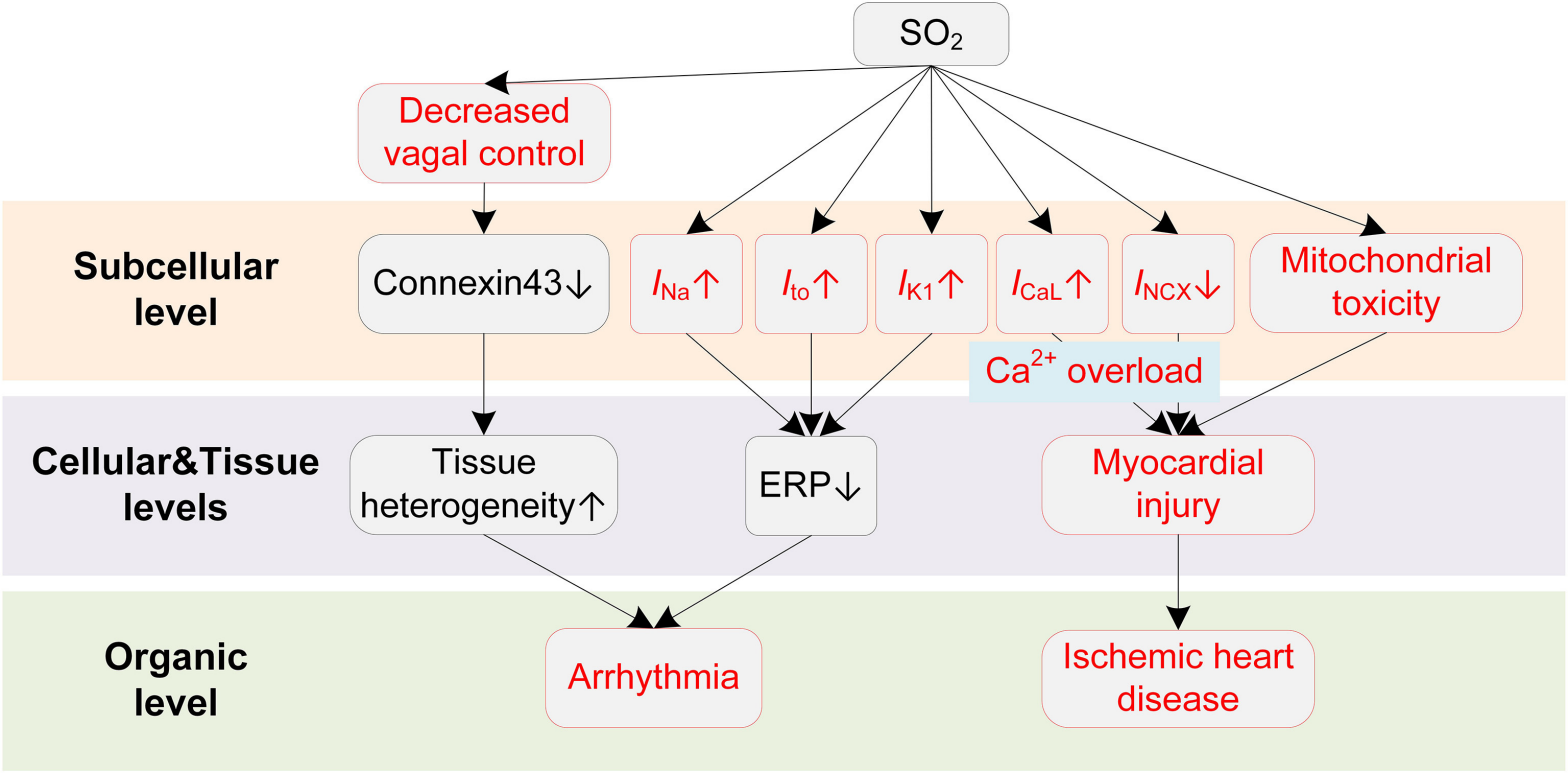
Potential mechanisms underlying SO_2_-induced arrhythmias. Red boxes indicate effects of SO_2_ that have been explicitly demonstrated.

SO_2_ can modulate various ion channels in cardiac myocytes, causing electrophysiological remodeling that predisposes to cardiac arrhythmias (Figure 5). Nie and Meng presented a series of experiments about the effects of SO_2_ derivations on ion channels in rat cardiac myocytes (154–156). In (154), the authors reported that the SO_2_ derivatives dose-dependently enhanced *I*_Na_ through a depolarizing shift of the inactivation curve. At a concentration of 10 μM, the half inactivation potential was shifted by 7 mV, causing an increase of ~42% in peak amplitude of *I*_Na_. Besides, the time courses of activation, inactivation, and recovery from inactivation were all accelerated (154). SO_2_ derivatives were also found to increase *I*_CaL_ in a dose-dependent manner (156). The activation and inactivation curves of *I*_CaL_ were shifted by 10 and 5 mV, respectively, toward depolarization direction, and the channel inactivation and recovery were accelerated (156). Consistent with above two studies, the *I*_NCX_ was inhibited by SO_2_ derivatives, and this was accompanied by the intracellular Ca^2+^ overload (157). To be more specific, the enhanced *I*_CaL_ leads to an increased intracellular Ca^2+^, which should be expelled via Na^+^/Ca^2+^ exchanger under physiological conditions. However, the expulsion is attenuated by the increased intracellular Na^+^ because of the SO2-induced increase in *I*_Na_. The accumulated Ca^2+^ eventually leads to irreversible myocardial damages, providing a possible mechanism for SO_2_-induced myocardial injury. For potassium channels, Nie and Meng reported that SO_2_ derivatives at 10 μM increased *I*_to_ and *I*_K1_ by 37.4% and 26.2, respectively. The enhancement was accompanied by hyperpolarizing shifted activation curve and depolarizing shifted inactivation curve and the shortened time courses (155). Similar to H_2_S, we investigated the proarrhythmic effects of SO_2_ using simulation approaches (158). Simulation results revealed that SO_2_-induced arrhythmogenesis may arise from several aspects. First, SO_2_ decreased APD and ERP, which generally facilitated the formulation of triggered activities and increased susceptibility to arrhythmias. As another consequence of the decreased ERP, the spatial vulnerability to reentry arrhythmia was increased in the SO_2_ affected tissue. The critical size for initiating reentry arrhythmia was shortened due to the decreased wavelength of spiral waves, which provided substrates for arrhythmogenesis. Third, the transmural repolarization dispersion was remodeled, and the decreased ΔERP attenuated the drift of the spiral wave and prolonged its lifespan (158).

SO_2_ also causes other cardiac dysfunctions that indirectly lead to arrhythmias. For example, a human challenge study presented direct evidence that SO_2_ could cause a decrease in cardiac vagal control (159). The vagal control reflects the input of the parasympathetic branch of the ANS to cardiac regulation (160), and it is classically thought to be antiarrhythmic by balancing the sympathetic stimulation and maintaining the gap junction function (i.e., preservation of phosphorylated connexin 43) in structural remodeled hearts (161). The decreased vagal activities, consequently, lead to the occurrence of arrhythmias. The systemic inflammatory response was excluded since no systemic acute phase or coagulant response was observed during exposure (159). Besides, several studies have suggested that SO_2_ could cause mitochondrial dysfunction and induce ROS production (162, 163), which provide possible underlying mechanisms for the significant association of SO_2_ to ischemic heart diseases. Above two pathological pathways for promoting arrhythmias have been discussed in previous sections and are not discussed here.

## Ozone

Ozone (O_3_) is a pale blue gas and has a very pungent odor. Ground-level O_3_ is formed primarily from photochemical reactions between volatile organic compounds and nitrogen oxides (164).

### Epidemiological and Clinical Reports

Epidemiological reports have provided substantial evidence on the associations of both short-term and long-term exposures to ambient O_3_ with adverse cardiovascular effects. For short-term effects, Buadong et al. reported that a 10 μg/m^3^ increase of O_3_ contributed to a 0.5% increase in the numbers of daily emergency hospital visits for cardiovascular diseases among aged individuals (165). Ensor et al. reported that the increase of O_3_ in the previous 1–3 h was associated with an increased cardiac arrest risk (166). An investigation conducted in Italy reported a direct and significant correlation between the number of daily ST-elevation myocardial infarction patients and the O_3_ concentration of the same day (167). On the other hand, Turner et al. reported significant positive associations between the long-term O_3_ exposure and cardiovascular mortality (168). The long-term influences were also observed in a recent cohort study in the United States, which reported significant associations of the long-term O_3_ exposure to cardiovascular diseases and ischemic heart diseases (169). For heart rhythm disorders, Rich et al. found a statistically significant association between ventricular arrhythmia and O_3_ exposure in 24 h before the arrhythmia (170). They also reported a positive association between episodes of paroxysmal AF and 1-h O_3_ exposure (171). The controlled human exposure to O_3_ reported three changes in cardiovascular systems, including an increase of inflammatory markers, changes in fibrinolytic markers, and changes in autonomic control of heart rate (172, 173). Prolonged QT intervals were also reported in both prospective follow-up studies and human exposure experiments, and such effect was immediately observed after exposure (121, 172).

### Potential Proarrhythmic Mechanisms

**Figure 6:**
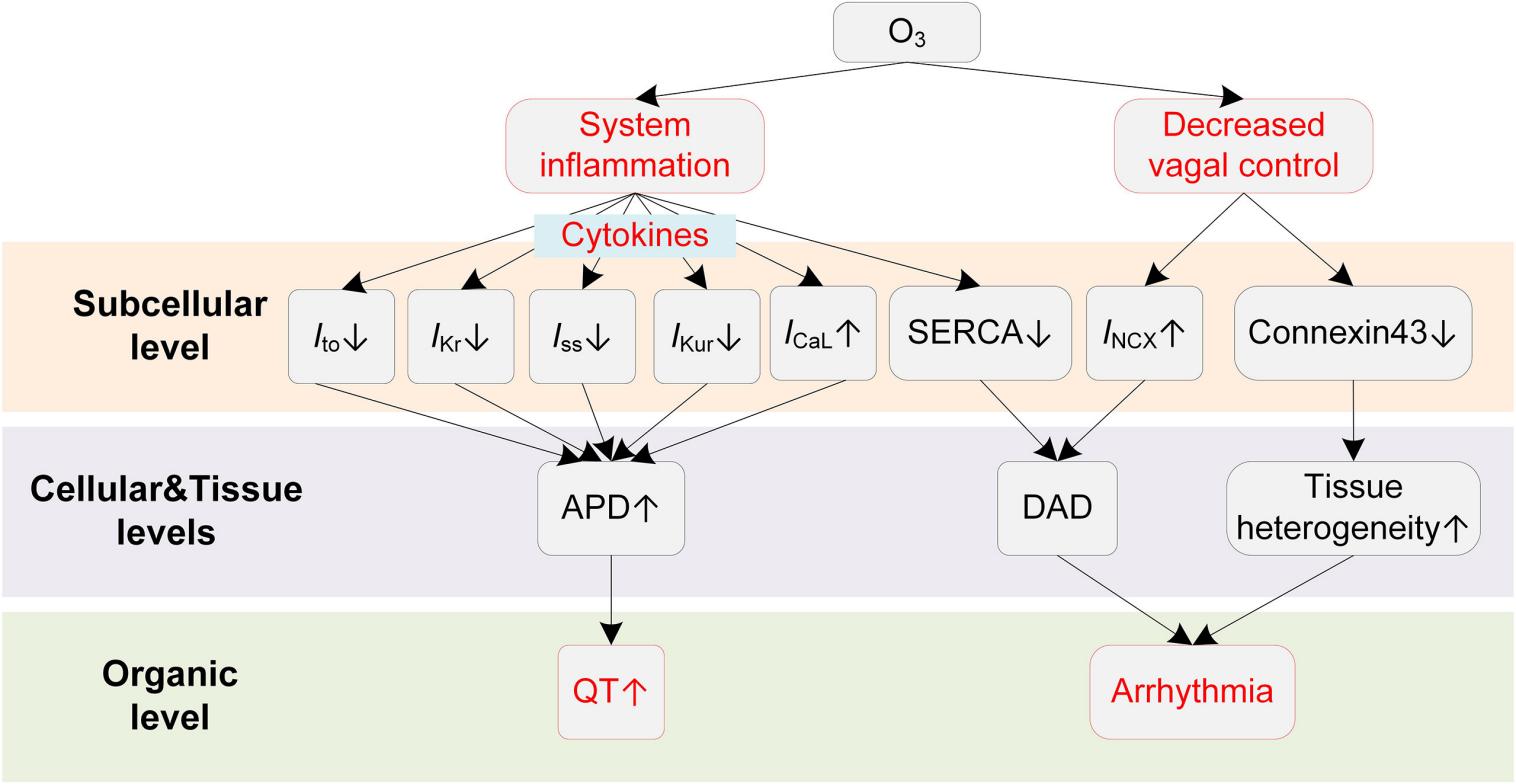
Potential mechanisms underlying O_3_-induced arrhythmias. Red boxes indicate effects of O_3_ that have been explicitly demonstrated.

Animal exposure studies have revealed that O_3_ could result in a series of changes in cardiac electrophysiology (Figure 6). Farraj et al. reported that exposure to O_3_ of 0.2 ppm was already able to increase myocardial susceptibility to arrhythmias, and a higher concentration (0.8 ppm) resulted in more rhythm abnormalities, including bradycardia, prolonged PR intervals, depressed ST segments, and frequent atrial premature beats and conduction blocks (174). The mechanistic investigation using a rat exposure model suggested that the inflammation played a major role in O_3_-induced arrhythmias (175). It was observed that acute O_3_ exposure led to paroxysmal VT in rats, and the changes of inflammatory cytokines such as tumor necrosis factor α (TNF-α) and interleukin 6 (IL-6) were greater than those of oxidative stress, e.g., superoxide dismutase (SOD) and serum malondialdehyde (MDA) (175). A recent clinical study demonstrated that systemic inflammation could lead to QTc prolongation via cytokine-mediated effects (48), and this finding provides new possible mechanisms for the O_3_-induced QT-prolonging observed in O_3_ exposure experiments. From a cellular perspective, the inflammation-induced QT prolongation arises from the alterations of multiple ion channels by cytokines. Specifically, TNF-α at physiologically relevant concentrations was recorded to reduce SERCA (45) and downregulate multiple repolarization currents in cardiomyocytes of rodent animals, including the transient outward potassium current (*I*_to_) (45, 176, 177), the steady state current (*I*_ss_) (45), and the ultra-rapid outward current (*I*_Kur_) (177). TNF-α was also reported to decrease *I*_Kr_ in the HEK293 cell (178). In addition to the TNF-α, other inflammatory cytokines, e.g., IL-1β and IL-6, could also lead to APD prolongation via decreasing *I*_to_ (179) or increasing *I*_CaL_ (180). Above alterations of ionic currents in the inflammatory condition together contribute to delayed repolarization of AP and manifest as the prolonged QT interval on the ECG.

ANS is also involved in the proarrhythmic effects of O_3_. The decreased HRV has been observed in both animal (175) and human exposure studies (172). As stated in previous sections, the decreased HRV implies weakened antiarrhythmic effects by the parasympathetic nervous system; it therefore acts as another proarrhythmic pathway after exposure to O_3_.

Currently, there is no experimental investigation showing the influence of O_3_ on cardiac ion channels. Therefore, the O_3_-induced subcellular electrophysiological changes are worth further study in the future.

## Nitrogen Dioxide

Nitrogen dioxide (NO_2_) is a reddish-brown gaseous pollutant with a pungent and irritating odor. It is formed primarily from high temperature combustion, and the prominent sources of NO_2_ include power plants, industrial furnaces and boilers, and motor vehicles (181).

### Epidemiological and Clinical Reports

Epidemiological studies have identified significant associations between short-term or long-term exposures to NO_2_ and hospital admissions for heart rhythm disorders and other cardiovascular diseases. An investigation conducted in the UK showed positive and statistically significant associations between short-term NO_2_ exposure and cardiovascular events, including an increase of 2.9% for arrhythmias (182). Similar results were reported worldwide, for example, in Spain (183), Belgium (184), and China (151, 152, 185). For the long-term NO_2_ exposures, an early epidemiological study showed that long-term exposure to NO_2_ had a significant effect on emergency room visits for arrhythmias (186). Such proarrhythmic effect of long-exposure to NO_2_ was supported by a recent investigation that traffic-relevant NO_2_ was in association with a higher risk of AF in both the young and middle-aged population (187). In addition, long-term NO_2_ exposure was also strongly associated with the risk of myocardial infarction (188).

Noted that an increasing amount of investigations propose the hypothesis that NO_2_ might be a surrogate for other pollutants (PM_2.5_) rather than directly influencing cardiovascular health (184, 189). The hypothesis is raised due to the same combustion emission sources of NO_2_ and other pollutants, but it is still disputable and not consistent in different studies. Seaton et al. first suggested a surrogate role of NO_2_, indicating that the toxicity of low concentration NO_2_ was just the result of confounding by PM (190). Scaife et al. also reported that NO_2_ failed to affect HRV in the absence of other pollutants (191). On the other hand, Samoli et al. reported an independent effect of NO_2_ on cardiovascular mortality (192). Besides, a national coverage study in the UK reported a strong NO_2_ effect with arrhythmias including AF and heart failure, and this strong effect persisted after adjusting PM_2.5_. In contrast, the PM_2.5_ effect was somewhat reduced when NO_2_ was adjusted (182). In summary, the underlying mechanisms for NO_2_-induced cardiovascular diseases, and the joint effects of NO_2_ and other pollutants deserve more in-depth investigations.

### Potential Proarrhythmic Mechanisms

**Figure 7:**
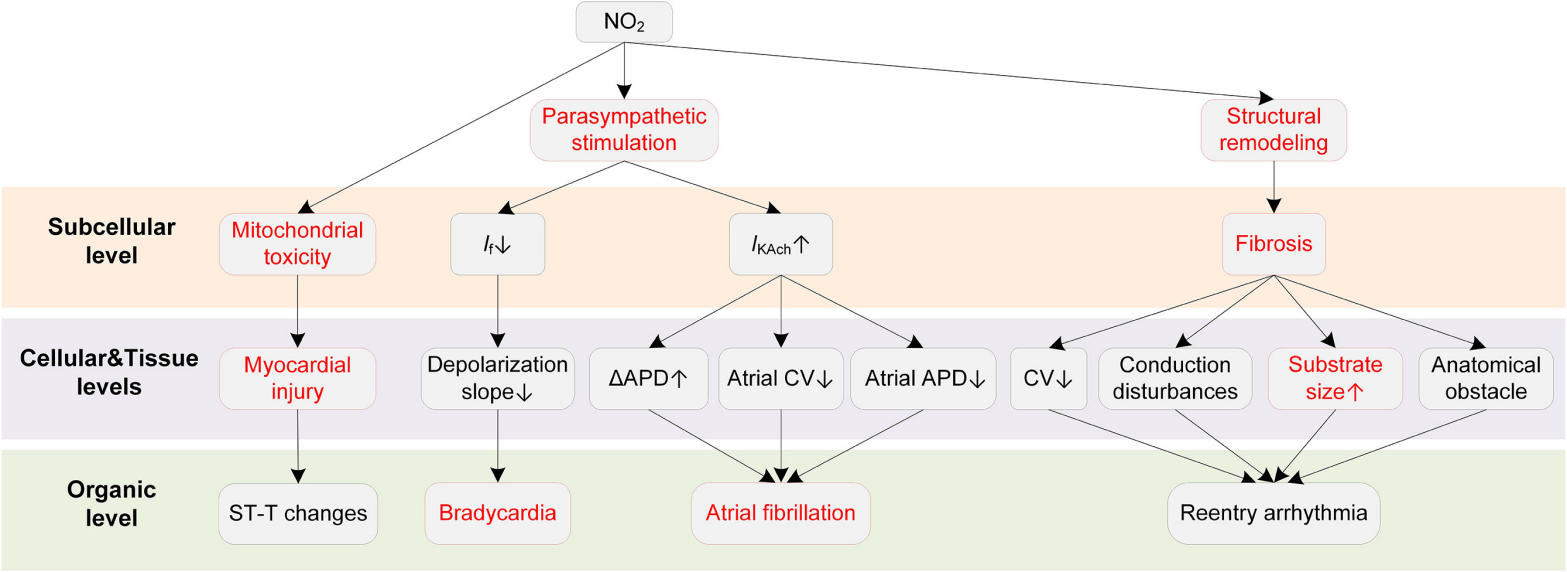
Potential mechanisms underlying NO_2_-induced arrhythmias. Red boxes indicate effects of NO_2_ that have been explicitly demonstrated.

Potential mechanisms of NO_2_-induced arrhythmias are summarized in Figure 7. Bradycardia is the major ECG abnormality after acute exposure to NO_2_, according to previous animal exposure experiments (193, 194). More severe arrhythmias were observed after 90 min to 3 h exposure, which were accompanied by atrioventricular blocks, premature beats, and wandering pacemakers. The severity of abnormalities was dependent on the NO_2_ concentration and exposure time (193).

The ANS is a critical pathway for NO_2_ to exert adverse cardiovascular effects. In an early animal investigation, exposure to NO_2_ led to bradycardia arrhythmias in healthy rats (193). However, the bradycardia was not observed in rats administered with the nitrite solution. The experimental results suggested that the elevated nitrite (NO_2_−) and nitrate (NO_3_−) in blood, despite being one of the consequences of the exposure to NO_2_, had no association with the arrhythmias caused by NO_2_. The study concluded that the changes in parasympathetic nervous activity were responsible for the proarrhythmic effects of NO_2_ (193). The involvement of ANS in the effects of NO_2_ was supported by some following studies (195, 196). From a cellular perspective, the NO_2_-induced parasympathetic stimulation decreases the *I*_f_ and the slope of phase 4 depolarization in the SAN and atrioventricular node (AVN), and finally slows the heart rate (197). The parasympathetic activation by NO_2_ may also contribute to atrial arrhythmia. It has been demonstrated that whether the parasympathetic stimulation is proarrhythmic or antiarrhythmic depends on the heart region (54). Specifically, the parasympathetic stimulation reduces ERP, augments the spatial electrophysiological heterogeneity, and promotes EAD particularly in the atria (54), whereas it prolongs APD and ERP in the ventricle (63, 64). These atria-specific effects provide a possible basis for the associations between NO_2_ and atrial arrhythmias that are frequently reported in epidemiological studies. In addition to the ANS, structural remodeling is another proarrhythmic factor of NO_2_. Exposure to a higher NO_2_ concentration was reported to be associated with larger biventricular volume in healthy individuals (87, 198). There were also studies considering NO_2_ and PM together as a joint proarrhythmic factor (182, 183).

As far as we are concerned, there are no studies investigating the alterations in ionic currents upon exposure to NO_2_. However, a recent study suggested that NO_2_ could impair the mitochondrial function under conditions of repeated exposures to NO_2_ at air pollution relevant levels (199). Mitochondrial dysfunctions including alterations of ATP synthesis and oxidative phosphorylation, and an increase in mitochondrial ROS production was observed after repeated NO_2_ exposures (15 h per week that lasted three weeks). In contrast, one-time acute exposure only induced moderate and reversible mitochondrial ROS production, and the ROS increase was not accompanied by mitochondrial alterations (199). The mitochondrial toxicity provides possible reasons for the myocardial infarction induced by the long-term exposure to NO_2_.

## Discussion

Air pollution is among the main factors in triggering cardiac arrhythmias. In this review, we focused on six common air pollutants, namely PM, CO, H_2_S, SO_2_, O_3_, and NO_2_, presented the epidemiological and clinical evidence of air pollution-induced arrhythmias in recent years, and analyzed the underlying mechanisms for each of the air pollutants. Based on the literature in this review, we summarized several areas that warrant further studies in the future.

First, confounding effects among pollutants should be examined at molecular and cellular levels, while epidemiological or exposure studies concerning individual air pollutant are needed. In detail, epidemiological studies have proved that some combinations of pollutants might enhance the toxicity of each other and lead to unexpected biological alterations; however, the corresponding cellular mechanisms remain unclear, with most basic medical research still focusing on a single factor, i.e., an individual pollutant or a specific component of PM. On the other hand, epidemiological or exposure studies with regard to a specific pollutant or PM component are relatively rare, which aggregates the mismatch of epidemiological evidence and mechanical findings at the cellular level. Since most epidemiological studies present the integral influence of various air pollutants, confounding research can be more persuasive in explaining complex mechanisms of pollution-induced arrhythmias.

Next, as illustrated in Figure 1, the pathological pathways for air pollution-induced arrhythmias can be interrelated or overlapped. For example, prolonged QT interval can be an integral effect of CO by enhancing *I*_NaL_, suppressing *I*_K1_, and subsequently prolonging the APD (114). However, such an effect may also arise as a consequence of systemic inflammation induced by O_3_, as the produced inflammatory cytokines contribute to electrical remodeling and prolong the QTc (48). Another example that has been frequently reported in relevant studies is the ST-T changes, including ST-segment elevation, ST-segment depression, and T wave inversion. The altered ST indicates impaired repolarization. It may arise from the tissue ischemia directly by some gaseous air pollutants, i.e., H_2_S and CO, but it can also result from the aggravated atherosclerosis by the PM_2.5_-induced systemic inflammation. In addition, the elevated ROS triggered by translocated PM can lead to myocyte apoptosis. These factors together lead to myocardial injury, generating an abnormal ST morphology. Due to the interrelated mechanisms, thorough investigations are always necessary before concluding proarrhythmic effects for a specific air pollutant. In contrast to the complicated and interrelated pathways, there are only a handful of experimental studies presenting the specific alterations in ionic currents upon exposure to air pollutants, which limits the summarized mechanisms in this review. Therefore, more fundamental studies are expected to elucidate the cellular electrophysiological effects of air pollutants.

Third, the computer simulation supplies an effective tool for integrating multiscale experimental data and for investigating the mechanisms underlying air pollution-induced arrhythmias. Mathematical models from the cell level to the tissue and organ levels have been extensively developed in past decades and have been successfully applied to investigate the mechanisms underlying congenital heart diseases (200), coronary heart diseases (42), and malignant ventricular arrhythmias (201), etc. Despite the sophisticated simulation measurements, the number of simulation studies regarding air pollution-induced cardiac arrhythmias is still quite scarce. In fact, the multiscale heart model has been proved to be a promising tool in investigating air pollution-induced arrhythmia. For instance, the proarrhythmic effects of CO, including the APD prolongation and the occurrence of EAD, were simulated using cardiac cell models by two separate groups (124, 202). Palacio et al. modeled the myocardial fibrosis caused by the PM_2.5_ in the human atria, which suggested the involvement of structural remodeling in the detrimental influences of PM_2.5_ (96). Recently, we conducted simulation studies regarding the proarrhythmic effects of H_2_S (43) and SO_2_ (158), and these two parallel studies explained important commonalities and differences of the adverse effects of sulfur-containing pollutants. Based on the physiological and the pathophysiological characteristics reflected by different levels of the multiscale heart model, these studies revealed the individual role of air pollutants in promoting arrhythmias and provided insightful mechanisms underlying electrophysiological dysfunctions.

In summary, although there has been a great deal of research focusing on air pollution-induced arrhythmias, studies regarding the effects of pollutants at the cellular level, especially the confounding effects, are relatively few. This significantly hinders the development in this area. Future investigations are encouraged to focus on the basic experimental research before associating pollutants with arrhythmia. The cardiac simulation is also a promising tool for investigating the air pollution-induced arrhythmia, as it provides multiscale insights into the pathological changes of electrophysiology at different levels. As a growing amount of data regarding interactions between air pollutants and cardiac ion channels become available, mechanisms underlying air pollution-induced arrhythmias are expected to be further revealed in computational simulations.

## Author Contributions

ZW and HZ conceived this study. SZ and WL drafted and edited the manuscript. All authors reviewed the final version of the manuscript.

## Funding

This work was supported by the National Key Research and Development Program of China (NO. 2018YFB0204204) and Shandong Postdoctoral Program for Innovative Talents (grantee SZ).

## Conflict of Interest

The authors declare that the research was conducted in the absence of any commercial or financial relationships that could be construed as a potential conflict of interest.

## Publisher's Note

All claims expressed in this article are solely those of the authors and do not necessarily represent those of their affiliated organizations, or those of the publisher, the editors and the reviewers. Any product that may be evaluated in this article, or claim that may be made by its manufacturer, is not guaranteed or endorsed by the publisher.

## Abbreviations

ΔAPD: Dispersion of repolarization
ADP: Adenosine diphosphate
AF: Atrial fibrillation
ANS: Autonomic nervous system
APD: Action potential duration
ATP: Adenosine triphosphate
AVN: Atrioventricular node
CaMKII: Ca^2+^/calmodulin-dependent kinase II
CICR: Calcium-induced calcium release
CO: Carbon monoxide
CV: Conduction velocity
CVD: Cardiovascular disease
DAD: Delayed afterdepolarization
EAD: Early afterdepolarization
ERP: Effective refractory period
H_2_S: Hydrogen sulfide
HEK293: Human embryonic kidney 293 cell
HRV: Heart rate variability
*I*_CaL_: L-type calcium current
*I*_f_: Funny channel current
*I*_K1_: Inwardly rectifying potassium channel
*I*_KATP_: ATP-sensitive potassium current
*I*_Kr_: Rapid delayed rectifier potassium current
*I*_Ks_: Slow delayed rectifier potassium current
*I*_Kur_: Ultra-repid outward current
IL-6: Interleukin 6
*I*_Na_: Sodium channel current
*I*_NaL_: Late sodium channel current
*I*_NCX_: Na^+^/Ca^2+^ exchanger current
*I*_to_: Transient outward potassium current
LQT: Long QT syndrome
MDA: Serum malondialdehyde
MI: Myocardial injury
NO_2_: Nitrogen dioxide
NO_2_−: Nitrite
NO_3_−: Nitrate
O_3_: Ozone
PKC: Protein kinase C
PM: Particulate matter
Pwd: P-wave dispersion
QTc: Corrected QT interval
QTd: QT dispersion
ROS: Reactive oxygen species
SAN: Sinoatrial node
SERCA: Sarco/endoplasimic reticulum Ca^2+^ ATPase
SO_2_: Sulfur dioxide
SOD: Superoxide dismutase
TdP: Torsade de Pointes
TDR: Transmural dispersion of repolarization
TNF: Tumor necrosis factor
VF: Ventricular fibrillation
VPC: Ventricular premature complex
VT: Ventricular tachycardia.
